# A deadly dance: the choreography of host–pathogen interactions, as revealed by single-cell technologies

**DOI:** 10.1038/s41467-018-06214-0

**Published:** 2018-11-06

**Authors:** Pratip K. Chattopadhyay, Mario Roederer, Diane L. Bolton

**Affiliations:** 1 Precision Immunology, NYU Langone Health, New York, 10016 NY USA; 2 ImmunoTechnology Section, Vaccine Research Center, NIAID, NIH, Bethesda, 20892 MD USA; 3 US Military HIV Research Program, Henry M. Jackson Foundation, Walter Reed Army Institute of Research, Silver Spring, 20910 MD USA

## Abstract

Pathogens have numerous mechanisms by which they replicate within a host, who in turn responds by developing innate and adaptive immune countermeasures to limit disease. The advent of high-content single-cell technologies has facilitated a greater understanding of the properties of host cells harboring infection, the host’s pathogen-specific immune responses, and the mechanisms pathogens have evolved to escape host control. Here we review these advances and argue for greater inclusion of higher resolution single-cell technologies into approaches for defining immune evasion mechanisms by pathogens.

## Abstract (web summary)

Diane Bolton and colleagues review the impact of single-cell technologies on the study of host–pathogen interaction. They discuss the revolutionary impact these have had for facilitating a greater understanding of the properties of host cells harbouring infection, the pathogen-specific immune responses, and the mechanisms pathogens have evolved to escape host control.

## Introduction

**Fig. 1:**
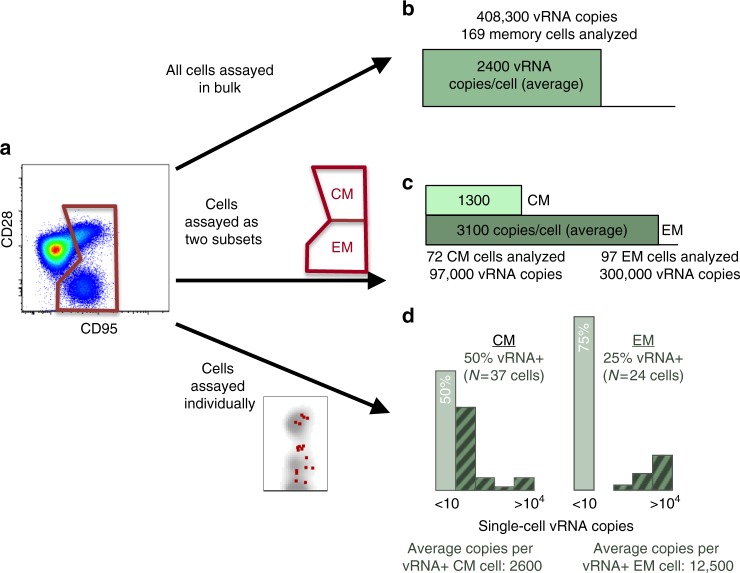
Single cell approaches provide finer resolution and more accurate analysis of host–pathogen interactions than bulk analysis. **a** Populations of cells can be defined by shared or distinct phenotypes (e.g., naive vs. memory (gated)). These populations of cells may differ in frequency of infection, and individual cells may differ in the number of pathogen transcripts expressed. These features of host–pathogen interactions can be ascertained with single-cell approaches. **b** Assay of all cells in bulk provides an inaccurate estimate of pathogen burden: there is no information about the frequency of infection and reports an average number of pathogen transcripts per cell (which does not reflect the actual number of transcripts in any of the cells assayed). **c** Sorting of cell populations (e.g., by fluorescence-activated cell sorting) can better resolve relative differences in pathogen burden between cell phenotypes (e.g., central and effector memory, CM and EM, respectively), but remains misleading in terms of infection frequency and number of transcripts. **d** Single-cell analyses (e.g., cell sorting of one cell per sample well) reveals differential infection frequencies and pathogen burden per cell between CM and EM cell populations. In this example, infected cell frequency in CM exceeds that of EM (50% vs. 25%), but infected EM cells harbor a larger per cell viral transcript burden (12,500 vRNA copies vs. 2600)

The relationship between pathogens and their hosts is complex and has been brought about by continuous co-evolution. A network of host cells and molecules attempts to survey, detect, and eliminate pathogens. In turn, pathogens have evolved elegant and varied approaches for evading host immune responses. By examining host–pathogen interactions, we aim to better understand the fundamental mechanisms involved in infection and immunity, and to provide a foundation for the rational design of new prophylactic or treatment strategies. With the recent emergence of sensitive single-cell technology platforms, host–pathogen interactions can be studied with a resolution and depth not previously possible (Box 1 and Fig. 1).

The choreography between pathogen, target host cells, and immune surveillance dictates the course of disease, and is likely to define transitions between acute, chronic, and latent infection, as well as transmission. Studying these interactions is complicated by changes to pathogen replication, persistence and resistance, throughout its life cycle. Many pathogens, such as malaria^1^, alter their host cell tropism during the course of disease, and others such as HIV^2^ and herpesviruses^3^ adopt latent infection states invisible to immune responses. Thus, preventing chronic and latent infections, immune evasion, and transmission requires an understanding of host–pathogen interactions at a finite level.

**Fig. 2:**
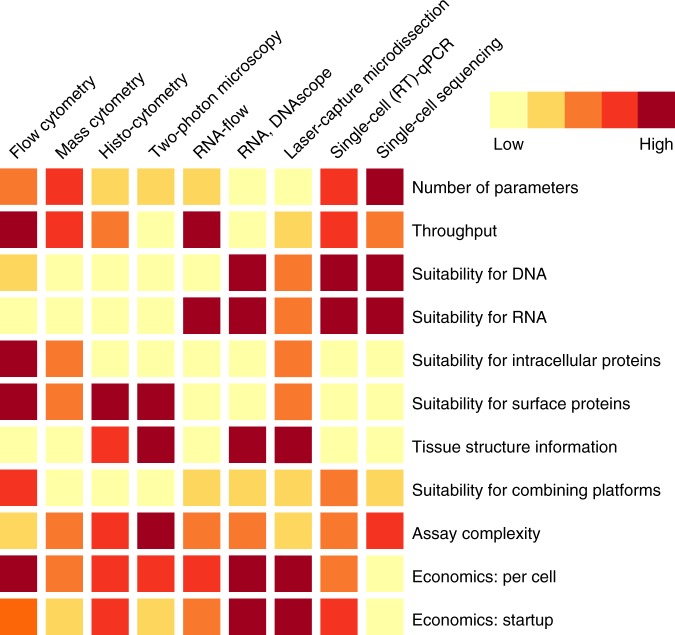
Heat map comparing various single-cell technology platforms. Relative capabilities of the single-cell technologies listed at top (columns) are depicted for each feature (rows). Performance is ranked from low to high relative to the other technologies using the indicated color scheme

Single-cell technologies relevant to the study of host–pathogen interactions are listed in Table 1 and Fig. 2 and a general overview of these methods is provided in Box 2. Using these platforms, significant advances have been made in understanding both successful and ineffective pathogen-specific immune responses, profiling pathogens, and understanding how host cell biology is affected by pathogen infection. For this, single-cell analyses have been invaluable. Here we review how the application of single-cell technologies has advanced our understanding of pathogen-specific immune responses, infected host cell profiles, and pathogen characteristics.

**Table 1:** Distinguishing features and underlying methods of single-cell technologies applied to the study of host–pathogen interactions

Platform	Description	Unique aspect	Value	Example application to host–pathogen studies	Host–pathogen theme(s) commonly addressed
Histocytometry	8-color single-cell imaging of antibody-stained fixed tissue.	Volumetric cell rendering and segmentation cleans images to identify single cells.	High parameter confocal, providing spatial context.	Increased T_FH_ cells in lymphoid tissue following vaccination^5^; CD8+ T-cell accumulation in lymph node germinal centers of HIV-1-infected individuals^4^.	Pathogen-specific immune responses
Two-photon intravital imaging	In vivo imaging of antibody-stained viable tissue.	Combination of infrared lasers and rare two-photon absorbance events focuses signal, allowing better resolution of single cells.	Deep tissue imaging with single-cell resolution and motility for live animals.	*L. major*-specific CD4+ T-cell deceleration at infection sites^10^; LCMV-specific T-cell exhaustion occurs via PD-1-induced motility paralysis^8^; *P. aeruginosa* infection sites recruit and direct neutrophils extravascular swarming via leukotriene B4^6^.	Pathogen-specific immune responses
High parameter flow, mass, or molecular cytometry	Cell suspensions stained with antibodies tagged with fluorescent dyes (flow), elemental isotopes (mass), or oligonucleotides (molecular).	High parameter analysis of protein expression at the single cell level.	Proteins mediate cell-to-cell interaction and extracellular communication, so their measurement provides more direct and accurate information than mRNA.	Studies of influenza vaccination and responses to CMV reveal the remarkable within and inter-individual variation in immune responses^14,15^.	Pathogen-specific immune responses
Fluorescence-activated cell sorting + Single-cell qPCR	Quantitative gene expression by PCR analysis of (c)DNA obtained from one cell; ~96 or more analytes.	Highly sensitive and robust quantitation of user-defined targeted panel of host and/or viral genes. Must be paired with single-cell capture device.	Multiplexing capability allows measurement of mRNA from multiple species. Targeted gene list limits multiple comparison penalty.	Rotavirus-infected and bystander intestinal epithelial cell interferon responses^39^; SIV and host gene expression profile of infected CD4+ T-cells^34^.	Infected cell profiling, Pathogen replication
RNA- and DNAscope	Hybridization based detection of pathogen nucleic acids in fixed tissue by microscope.	One portion of probe binds pathogen target, while other side is used for signal amplification. Complementary probes, each with fluorescent or enzymatic tags, are layered stepwise for signal amplification.	Allows extensive signal amplification.	CMV infection of intestinal epithelial cells and tight junction disruption independent of HIV-1^26^; Localization of HIV-1 and SIV RNA or DNA+ cells across and within tissues, including burden within single cell^25,45^.	Infected cell profiling, Pathogen replication
RNA-flow	Detection of pathogen nucleic acids by flow cytometry.	Like RNA- and DNAscope, but with flow cytometry-based read out.	Can be coupled with measures of protein expression to better identify cells, throughput of flow cytometry-based assay.	Co-expression of HIV-1 RNA and protein used to characterize infected patient cells^30,31,65^; Yellow fever virus RNA+ cell identification^28^; Kaposi sarcoma-associated herpesvirus augments Epstein-Barr virus tumorigenesis in dual-infected cells^29^.	Infected cell profiling
Single-cell RNA sequencing	Whole transcriptome analysis or targeted sequencing of 400–800 mRNA.	Unbiased full transcriptome; typically coupled with single-cell capture device.	Of all technologies, provides information on the highest number of parameters.	Reactivated latent HIV-1-infected CD4+ T-cells express virus-silencing genes^37^; Subpopulations of *S. typhimurium*-infected macrophages suggest linear, consecutive stages of infection^59^.	Infected cell profiling, Pathogen replication
Laser-capture microdissection	Isolates one cell from a microscopic region of interest.	Capture methodology for cell does not disrupt tissue.	Allows study of single cells from a region of interest in tissue.	HSV-1 and varicella zoster virus DNA persistence in sensory neurons^48^; HIV-1 provirus burden in patient splenocytes^52^.	Infected cell profiling, Pathogen replication

### Box 1 Advantages of single-cell analyses over bulk measurements

Single-cell measurements are defined as quantitation of one or more analytes (e.g., RNA, DNA, protein) associated with a single cell. Depending on the method, the assay may be performed on a cell residing within a tissue section or in suspension. Single cell-based approaches applied to the study of hosts and pathogens offer important advantages over bulk cell analysis (Fig. 1). The latter often relies on a mix of infected and uninfected cells and responding and nonresponding cells. Thus, signal from uninfected or non-specific cells dilutes information about host–pathogen interactions. In some cases, critical proteins associated with the cells of interest may be diluted below the limit of detection, while background signal from uninvolved cells increases. This is a particularly impactful problem, because pathogens are typically small (with much lower protein, DNA, and RNA content than cells) and because infected cells are typically rare. Bulk analysis also averages information about protein or transcript expression, so that precise cell-by-cell phenotypes cannot be assessed. Knowledge of the specific phenotype of infected cells, or responding immune cells, may help reveal mechanisms of infection, immune evasion, or successful immunity. Perhaps most important is that bulk analysis provides no information about the coordinate expression of multiple molecules in individual cells. At a minimum, this occludes the ongoing communication occurring between pathogen and host, and amongst cells in the host immune system. Despite these limitations, comparisons of bulk to single-cell measurements should be performed during the initial evaluation of any single-cell technology, in order to demonstrate that there is no loss of assay sensitivity at the single-cell level. An approach to performing single-cell/bulk comparisons is demonstrated for highly multiplexed qPCR by Dominguez et al.^64^.

### Box 2 Overview of single-cell technologies and platforms

Single-cell technologies are now available for a wide array of settings, including tissue and cell suspensions, in vitro and in vivo studies, and mRNA and protein analysis. In this article, we review imaging platforms (like histocytometry and two-photon imaging), high parameter cytometry-based technologies (like fluorescence flow cytometry and mass cytometry), in situ hybridization approaches (like RNAscope and PrimeFlow), and single-cell molecular platforms (like highly multiplexed single cell qPCR and RNA-seq). Beyond these platforms, there are many new extraordinarily sensitive and exciting technologies that are being applied to single-cell analyses, such as super-resolution imaging, in situ PCR analyses, introduction of multiple fluorescent reporters at key genetic sites in cells or animals. These will certainly inform host–pathogen (and host-cancer) interactions in the future, but have not been broadly applied as yet. Thus, those technologies are not covered here, but we hope that the successes reviewed here will spur the application of such technologies to this subject. In addition, we focus primarily on studies employing intact pathogens in human or animal models of infection, with less emphasis on studies involving pathogen components (e.g., nucleic acid or pathogen-associated molecular pattern compounds) and in vitro tissue culture models. Whole organism studies are more likely to capture the full range of complex interactions between host and pathogen.

## Pathogen-specific immune responses

Immune responses are inherently complex, involving many different functional types of cells, and often occur in highly organized tissue microenvironments. Technologies that visualise the three dimensional organizations of host responses to infection, and those that enable the isolation of cells for further analyses, have advanced our understanding of how pathogens influence host immune responses. Both approaches enable the simultaneous measurement of multiple parameters, revealing how immune responses are initiated and coordinated in the tissue microenvironment, and they facilitate in-depth characterization of transcripts or protein in both natural infection and vaccine settings.

### Histocytometry

Imaging technologies, such as histocytometry (see Table 1), address fundamental questions in immunology, such as how human T-cells provide helper signals for antibody-secreting B-cells. To date, a major limitation to the study of this process has been the lack of readily available draining lymph nodes, which is the tissue where these interactions are thought to occur. This is primarily because these tissues are not routinely sampled after pathogen exposure, and the often invasive nature required to obtain samples; indeed, the gut- and lung-associated lymph nodes require surgery for sampling.

Understanding the phenotype, function, and localization of CD8+ T-cells within lymph nodes has important implications for the eradication of chronic viral infections, even in treated individuals. During HIV infections, lymph nodes, particularly their germinal centers and follicles, are an active site of viral replication and re-activation from latency, and CD8+ T-cells play an important role in killing HIV-infected cells. In 2017, Petrovas et al. used histocytometry to study CD8+ T-cells in the lymph nodes of HIV-infected individuals^4^. The authors showed that CXCR5^hi^ CD8+ T-cells accumulate in germinal centers, demarcated by co-expression of CD20 and Ki67. Detailed characterization of the heterogeneity in the differentiation and checkpoint markers within these cells showed that they are capable of potent cytolytic function. Furthermore, these can be directed to kill HIV-infected cells using bi-specific antibodies that bind HIV proteins on the cell surface and CD3 on the surface of the follicular CD8+ T-cell. In this particular example, histocytometry facilitated the mapping of these complex cellular phenotypes to lymph node germinal centers at single-cell resolution.

Recently, histocytometry has been used to show that tonsil tissues obtained from children contain an organized microanatomy that is typical of secondary, draining lymph nodes^5^. Of note, the tonsils studied contained clear germinal center and follicle structures. Within these structures, follicular helper T-cells (T_FH_) have been shown to provide key signals for the proliferation, differentiation, somatic hypermutation, and isotype-switching of B-cells. Thus, tonsil tissue was confirmed as a model for draining lymph nodes. Amodio et al. next studied responses to seasonal influenza vaccine within the tonsil tissue microenvironment^5^, finding higher levels of T_FH_ cells in vaccinated compared to unvaccinated children. Notably, regulatory cells that suppress T_FH_ were localized to extrafollicular areas of the tonsil, and these cells were diminished in vaccinated children. Cell subsets were profiled for a number of other markers in the study, providing multidimensional information about the organization and activity of the helper T-cell response after vaccination. In sum, this study demonstrated that a readily accessible tissue could provide an important model for how immune responses are staged in lymph nodes.

### In vivo imaging

Although powerful, a limitation of histocytometry is that it only provides a snapshot of an immune response at a single, static time point. Immune responses are dynamic as cells are recruited, engage the pathogen or infected cells, and then return to unactivated or memory states. The study of these dynamic aspects requires in vivo imaging technologies such as two-photon imaging (see Table 1). The remarkable resolution of two-photon imaging enables location information to be provided at both the broader tissue level and within tens of microns around single cells.

In 2013, two-photon intravital imaging revealed dense clusters of neutrophils that rearrange local collagen networks in tissues, following infection with *Pseudomonas aeruginosa*, which leads to better access to the pathogen by host immune cells^6^. Upon infection of a mouse with *P. aeruginosa*, neutrophils are recruited by the lipid leukotriene B4, which is released by dying host cells at the site of infection. In this way, leukotriene B4 amplifies “danger” signals, increasing the distance from which neutrophils can be recruited. Furthermore, two-photon intravital imaging identified an important role for the integrin receptor in maintaining these neutrophil clusters, and the rearrangement of collagen networks. Prior to this, integrin receptors had not been associated with this cell-to-cell contact function in host–defense; they were thought to be dispensable for neutrophil migration.

The ability to contextualize single-cell data represents an important virtue of imaging technologies—and underscores limitations of studying the immune system in cells removed from these contexts. In an example from 2012, Muller et al. demonstrated that CD4+ T-cells specific for *Leishmania major* had direct contact with only a minority of infected cells^7^. This was surprising as it showed the immune system honing in on specific pathogen-infected cells; however, significantly, the authors found that IFNγ, secreted as a consequence of this relatively rare immune:infected cell interaction, was distributed across a gradient up to 80 μm away from the immunological synapse. This remarkable finding suggests the promotion of a bystander effect, whereby intracellular defense mechanisms are triggered in neighboring cells despite minimal initial immune:infected cell interaction.

Single-cell imaging also identifies changes in immune cell behavior after exposure to pathogens. For persistent pathogens, two-photon imaging identified changes that lead to anergized T-cells. In a lymphocytic choriomeningitis virus (LCMV) model of chronic infection, two-photon imaging showed that the motility of LCMV-specific CD4+ and CD8+ T-cells is reduced within the lymph node of chronically infected animals, and that this “motility paralysis” is facilitated by the binding of the exhaustion molecule, PD1, to its ligand, PDL1^8^. Similarly, in *L. major*, within minutes after exposure intradermal dendritic cells became immobilized^9^. However, unlike the exhausted T-cells in the LCMV model, immobilized dendritic cells were thought to still participate in immune responses because they are able to phagocytose *L. major* efficiently. However, subsequent studies showed that the immune response to *L. major* is limited in a variety of ways, across different parts of the infected tissue. Despite phagocytic activity of immobilized dendritic cells, in some tissue regions, dendritic cells carrying *L. major* accumulated and left little access for T-cells^10^. In other tissue regions, CD4+ T-cells interact with infected phagocytes but these interactions are often short-lived, suggesting suboptimal or inefficient immune responses. A similar immune arrest phenomenon may occur during *Mycobacteria* infection, where T-cells within the granuloma only weakly produce cytokines in vivo, despite exhibiting robust cytokine production in vitro^11^.

Taken together, these studies show that pathogens can “dance around” immune responses in complex ways, and that high parameter tissue imaging of single cells is well suited to define the steps in this dance.

### High parameter, high throughput single-cell technology

Immune responses to pathogens involve a variety of cells; often, specific subsets of cells within both innate and adaptive arms of immunity are invoked. Specific functions of responding immune cells may be diverse, for example multiple cytokines can be expressed simultaneously or in specific combinations; such finite characterization can only be done using high parameter single-cell technologies.

High parameter, high throughput technologies such as flow cytometry, qPCR, and RNA-seq (see Table 1) enable broad investigation and characterization of immune lineages, and the functions of all cells involved in a response. Such technologies revealed how host-responding cells change over the course of an immune response, differ amongst individuals, and even change with circadian cycles^12^.

### Flow cytometry

Both fluorescence-activated cell sorting (FACS) and mass spectrometry-based cytometry (CyTOF) are used to interrogate immune responses^13^. There are advantages to either technique. FACS allows high-throughput analysis, compatibility with a large body of reagents, and the ability to isolate individual or large numbers of viable cells for further profiling. CyTOF allows ease of multiparameter antibody panel development, and a higher level of multiplexing.

High parameter cytometry approaches have been used to assess multiple aspects of cell phenotype and function, including differentiation state, proliferation potential, trafficking, cytotoxic capacity, and cytokine secretion. Notably, many of these traits are co-expressed in combinations that can define, very precisely, cellular states^13^. Over the past decade, a number of platforms based on detection of fluorescence, elemental isotopic, or molecular signals have emerged for high parameter analysis. These powerful technologies have been used recently for improved characterization of human immune responses and their variation. In 2014, individuals were studied before and after vaccination for seasonal influenza using polychromatic flow cytometry^14^. There was remarkable inter-individual variation in cell frequencies before vaccination, with evidence of remodeling of the peripheral immune compartment following vaccination, including the upregulation of hundreds of transcripts. Most importantly, baseline pre-vaccination immune cell populations could predict antibody titers post-vaccination, suggesting that immunophenotyping of healthy individuals before vaccination could be used to predict vaccine efficacy. Indeed, this concept was suggested in 2015 in a study of immune parameters in twins using both fluorescence flow and mass cytometry^15^; 58% of the measured immune traits were determined by non-heritable factors. This trend was similar to that seen for immune parameters following vaccination; antibody responses to seasonal influenza vaccination were driven by non-heritable influences, and more than half of immune parameters in twins discordant for cytomegalovirus (CMV) exposure differed. These results demonstrate how single-cell technologies can reveal intricate heterogeneity in immune responses to vaccination, and can help to understand the genetic basis and predictive power of finely defined subsets.

### Single-cell transcriptomics

Single-cell transcriptomics enables the quantification of mRNA from individual cells. Both qPCR and sequencing measure RNA from multiple single cells at the same time, which provides quantitative coordinated gene expression data. Multiplexed qPCR was an early approach to single-cell gene expression analysis and targets a specific set of genes, typically pre-defined by the user. However, as the economics and sensitivity of single-cell mRNA sequencing (scRNA-seq) improved, it has increasingly substituted qPCR, as it allows quantification of many more mRNAs and in an unbiased manner.

Indeed, an early approach for deep profiling of immune cells combined FACS with highly multiplexed single-cell qPCR^16^. Single cells defined by expression of a dozen or more proteins were isolated, and then quantified for the expression of a minimum of 96 mRNA transcripts (see Table 1). In 2011, this approach was used to identify mechanisms of protection by vaccines; specifically, the comparison of HIV-specific T-cells generated by three different vaccine regimens: (1) DNA vaccine prime/recombinant adenoviral vaccine boost, (2) recombinant adenoviral prime and boost, and (3) recombinant adenoviral prime/recombinant LCMV boost^16^. When assayed by standard flow cytometry, vaccine-specific cells were indistinguishable across the three regimens in terms of frequency, phenotype, or functionality. However, 96-parameter mRNA analysis distinguished the vaccine regimens, and identified regimen-specific gene signatures. Notably, the regimen using a DNA prime elicited more central memory-like than effector memory-like T-cells.

The power of scRNA-seq was used to show that antigen-induced cells functionally adapt to local tissue environments to stop the spread of a pathogen throughout the organism based on finite profiling of genes specific to lung vs. liver T-cells^17^. In contrast, another approach showed how local reprogramming of cells can help spread infection with varicella zoster virus (VZV)^18^. This is counterintuitive to how we understand the immune response, but evidence from tonsil tissues infected with VZV has shown that the virus can infect various types of T-cells, and that infection reprogrammes a variety of cells to express markers for skin homing, thus driving infected T-cells to propagate infection in the skin and likely aid virus transmission.

### High parameter single-cell imaging

Two recent technologies have adapted high parameter approaches to imaging platforms in remarkable ways. The first, known as Imaging Mass Cytometry (IMC), employs lanthanide-tagged antibodies, similar to suspension mass cytometry, but using laser ablation to collect cell staining signals; the resulting cloud of lanthanides at each location in the tissue is collected and transferred to a standard CyTOF instrument^19^. Although this approach has not yet been applied to profiling immune responses or pathogen-infected cells, it is likely possible, given a recent report describing combined analysis of mRNA and protein expression in tissue samples from breast cancer^20^. The second platform, known as CODEX, uses oligonucleotide-conjugated antibodies, which are then detected by hybridization of a complementary probe conjugated to one of three fluorescent reporters^21^. The fluorescent reporters can be quenched, allowing multiple rounds of triplexed detection of antibody targets. The current technology allows approximately 50-parameter analysis, and provides the spatial context that is a common virtue of all imaging approaches. Both technologies are promising, however, currently their throughput does not compare favorably to suspension-based cytometry technologies.

## Profiling infected cells

To understand the pathogenesis of an infectious disease, it is important to study the effects of infection on target cells to understand how they initiate the immune response and possible pathogenesis. Phenotypic and functional analysis of these cells requires technologies that can distinguish infected from uninfected cells. Notably, this is a difficult endeavor: if the cells were easily identifiable, the pathogen would not survive the ensuing immune response.

The primary markers used to identify infected cells are microbe-specific proteins and nucleic acids, including those embedded in the cell membrane such as viral glycoproteins, intracellular proteins such as viral capsid or proteins that regulate replication, and pathogen RNA or DNA. These microbial elements may be labeled using antibodies or oligonucleotide probes conjugated to fluorochromes or enzymes, which are applied to specimens directly ex vivo or from in vitro assays. By combining measurements of one or more microbial-specific tags with host cell phenotyping assays, the effect of pathogens on host cells, or their cellular preferences, can be profiled at the single-cell level. The major advances in profiling infected cells include a greater understanding of where they reside in vivo and describing their phenotype and molecular profile.

A major challenge in the study of many infectious diseases is identifying where pathogens replicate in vivo. Locating infected cells and linking them to transmission events, pathogenesis, and persistence is important for prophylactic and therapeutic strategies aiming to co-localize anti-pathogen agents, such as immune responses and drugs, at replication sites. Studying the location of infected cells requires tissue imaging approaches capable of much higher throughput compared to classic microscopy platforms. High-throughput imaging is particularly valuable for screening rare populations of infected cells within clinical or preclinical specimens, like HIV-1-infected cells. Identification of these cells is not only challenging because of their paucity, but also because the reagents available to uniquely and specifically identify them are poor. One recent example that substantially advanced our understanding of HIV transmission used a novel luciferase reporter virus and high-throughput imaging to find small foci of simian immunodeficiency virus (SIV) replication within vast tissue sections^22^. Shortly after vaginal challenge with SIV, infectious foci were identified throughout the entire female reproductive tract, including the ovaries. In addition, specific immune cell types, including Th17 CCR6+ CD4+ T-cells, within these tissues were preferentially infected, and may represent the portal of entry of HIV in human mucosal infection. These findings point to widespread SIV/HIV access and susceptibility within reproductive tissue during mucosal transmission.

### In-situ detection of pathogen genomes

Elucidating the spatial relationship between infected cells and disease-related pathologies is also a major challenge, but is essential for understanding pathogenesis processes in vivo. To locate infected cells in situ, improved hybridization technologies are now capable of easier and faster detection of cell-associated pathogen nucleic acid. New methods, known as RNAscope and DNAscope, use oligonucleotide “Z” probes against target sequences as a substrate for “branched signal amplification.” The approach maximizes signal and limits background^23–25^, achieving single-molecule sensitivity in some cases. Application of DNAscope to the study of CMV infection implicated infected cells in the disruption of intestinal barriers in HIV-infected individuals. Maidji et al. detected cells positive for CMV DNA in rectosigmoid tissue of asymptomatic CMV-positive individuals, and these CMV-infected cells were proximal to intestinal tissue integrity loss^26^. The ability to identify infected cells at the single-cell level, and simultaneously co-localize them to sites of tissue damage revealed an important role for CMV DNA+ cells in mediating disease during HIV-1 and CMV co-infection. These findings highlight a key advantage of microscopic approaches to understanding positional pathogen-mediated host effects.

For diseases that exhibit latent stages of infection in which viral replication is suppressed by stochastic or active mechanisms, such as therapy and adaptive immunity, there is great need for locating and characterizing the cells that harbor dormant pathogen in vivo. Combining RNA- and DNAscope methods permits the identification of these latent, transcriptionally inactive infected cells, which are characterized by the presence of viral DNA (vDNA) and lack of vRNA. This duplexed approach was successfully used to study lymph nodes from chronically SIV-infected rhesus macaques, revealing vDNA+vRNA– (latent) and vDNA+vRNA+ (active) cells^25^. In addition, quantifying SIV DNA+ and RNA+ cells before and after suppressive antiviral therapy has revealed disproportionate persistence of infected cells in B cell follicles relative to T-cell zones. Thus our ability to define the infection status of single cells and their location within tissues during treatment and disease has revealed potential havens that preferentially shelter HIV-1-infected cells. Further multiplexing with antibodies or other probes against host molecules will provide insight into regulatory factors and biomarkers of latent HIV-1/SIV cellular infection in vivo.

### Flow cytometric detection of pathogen nucleic acids

Efforts to eradicate infected cells, such as HIV “cure” efforts, also require a better understanding of their phenotype and molecular profile. What host (and pathogen) proteins are present or lacking on the cell surface and do they represent unique biomarkers that can be employed in strategies to target cells for elimination? To answer these questions, methods must identify infected cells with high specificity while accurately assessing host protein expression. Emergence of methods to measure mRNA by flow and mass cytometry show particular promise for identifying and characterizing infected cells. Fluorescence cytometry-based methods include flow-fluorescence in situ hybridization (flow-FISH), PrimeFlow, or RNA-flow, while the proximity ligation assay for RNA (PLAYR) method has been developed for mass cytometry^27^. This technology has been applied to the study of cells infected with yellow fever virus (YFV), Epstein-Barr virus (EBV), Kaposi sarcoma-associated herpesvirus (KSHV), and HIV. In the case of YFV, multiplexed staining with probes specific for negative and positive strand YFV RNA in humanized mouse models identified new immune cell subsets permissive to viral replication^28^. Moreover, the outcome of YFV infection and the generation of a protective immune response are both associated with the dynamics of infection across these cell subsets. In a mouse model of EBV/KSHV dual infection, flow-FISH specific for EBV RNA was used to demonstrate collaboration between these viruses^29^. Specifically, the majority of KSHV-infected splenocytes were EBV RNA-positive, implicating EBV-transformed cells as a primary KSHV target which ultimately facilitate KSHV persistence and associated tumor formation. Lastly, characterization of HIV RNA-positive CD4 T-cells in PBMC revealed that this transcriptionally active subset of infected cells exhibits markers of exhaustion. Moreover, these cells express markers of peripheral follicular effector memory helper cells^30,31^. Development of combined RNA- and DNA-flow will enable phenotyping of DNA-positive RNA-negative latently infected cells, described above. Study of these cells could lead to novel curative anti-HIV therapies. These examples illustrate the power of flow cytometric viral nucleic acid detection to not only identify and phenotype infected cells from human or animal specimens, but also link disease outcomes with cells exhibiting a distinct viral RNA profile.

Similarly, investigation of immunomodulatory and immune evasion strategies benefit from a range of single-cell methods, including microscopy, flow cytometry, PrimeFlow, and fiber-optic array scanning technology. Recently, multiple groups have confirmed that in vivo HIV/SIV-infected CD4+ T-cells exhibit reduced or negative CD4 surface expression^22,30–34^, consistent with in vitro results. For SIV, CD3 downregulation was also apparent^22,32,34^. Evidence of surface MHC class I modulation, however, was not readily or consistently observed on SIV-infected cells^34,35^, even though it has been reported in vitro. It will be important to explore these and other immunomodulatory strategies with other approaches directly ex vivo to determine the extent to which such processes occur during infection with HIV and other pathogens. Given the importance of CD3, CD4, and MHC class I molecules in processes including T-cell activation, viral entry and antigen presentation, these findings have important implications for our understanding of in vivo SIV and HIV-1 immune responses and replication.

### Single-cell transcriptomics

In order to define gene regulatory processes induced, or selected for, by pathogens to foster a favorable cellular environment for replication, we must interrogate gene expression. Defining the transcriptional profile of infected cells will reveal novel expression patterns underlying key aspects of the pathogen life cycle, which then can be exploited for drug or vaccine development. For example, host genes differentially regulated in infected cells would indicate cellular factors that support or inhibit pathogen replication. scRNA-seq represents the most powerful frontier for assessing the molecular properties of infected cells.

Pathogens investigated by host cell scRNA-seq to date include HIV and Zika virus (ZIKV), while a more focused qPCR approach was used for rotavirus and SIV. Studies of host genes associated with HIV-1 permissiveness yielded expression profiles that correlate with in vitro susceptibility to HIV infection^36^. To identify genes associated with latent HIV-1 infection, scRNA-seq of ex vivo reactivated HIV-1+ cells revealed elevated levels of HIV-1 suppressive genes and decreased type I interferon (IFN) responsive genes relative to control uninfected cells^37^. Potential host cell targets for ZIKV infection were identified in the human nervous system using scRNA-seq to quantify expression of candidate flavivirus entry receptors among cortex primary cells^38^. Lastly, 96-multiplexed qPCR analysis of infected and bystander cells has revealed how rotaviruses and SIV evade host antiviral innate responses and cause disease; type I IFN induction plays a key role in viral replication in infected host cell subsets^34,39^. Molecular profiling has thus advanced knowledge of cell types targeted by viral infection as well as host genes involved in ZIKV, rotavirus, and HIV-1 replication.

### Emerging technologies: single-cell multi-omic profiling

Finally, pathogen-mediated changes to infected cell phenotypes are accomplished by a myriad of molecular mechanisms including transcriptional, post-transcriptional, and post-translational processes. Single-cell methods that combine both gene and protein expression enable the study of these mechanisms directly within infected cells. For example, just as flow cytometry can be combined with highly multiplexed qPCR to examine host immune responses, the two platforms can be piggybacked for analysis of infected cells. For each cell analyzed, protein and mRNA expression data can be quantified simultaneously to reveal post-transcriptional regulation at the single-cell level. This assay relies on “indexed” cell sorting, first developed in the Herzenberg laboratory at Stanford in the 1980’s (but the power of which has only recently become widely employed^40^; for example, see ref. ^41^). Cells are sorted by fluorescence into individual wells of a PCR plate for lysis and mRNA quantitation, with a record of the complete phenotype of each cell stored for correlation against the subsequent assays. This approach was recently used to phenotype cells infected with SIV in vivo, identifying SIV RNA-positive cells by RT-qPCR for viral transcripts and then examining expression of host mRNA and proteins within these cells^34^. Exploring the mechanism of CD4 downregulation, *CD4* transcript levels were unchanged in infected cells with diminished surface CD4 protein, suggesting that post-transcriptional regulation of CD4 occurs in vivo, just as it occurs in vitro. In addition, the downregulation occurred in cells expressing higher levels of spliced viral RNA, consistent with downmodulation mediated by viral proteins encoded by spliced transcripts. Thus by simultaneously quantifying single-cell viral gene, host gene, and host protein expression, we have a greater understanding of the molecular mechanisms by which AIDS viruses alter host protein and transcriptional profiles of infected cells in vivo.

These studies of cells in suspension share a common theme. High parameter, single-cell technologies revealed the complex heterogeneity of host responses or infected cells, which express different genes and proteins depending on vaccine regimen, tissue, or infection status. In coming years, studies relating the various cell types to disease outcome will increasingly be performed using newly developed, high sensitivity 30+ parameter flow cytometry and cell sorting or Integrated Molecular Cytometry Platforms (IMCPs). IMCPs, like cellular indexing of transcriptomes and epitopes by sequencing (CITE-seq, Ab-seq) and RNA expression and protein sequencing (REAP-seq), use oligonucleotide-tagged antibodies to measure protein expression on a single-cell sequencing platform, providing (for each of up to 10,000 cells) whole transcriptome information simultaneously^42,43^.

Notably, these technologies underscore limitations of RNA-based analyses of cells: the differing half-lives of mRNA vs. their encoded proteins, as well as post-translational modifications, intracellular trafficking, and co-localization of proteins can all impact the functionality of cells that nominally have the same level of mRNAs. While scRNA-seq is indeed powerful, it is important to remember that all cell-cell and extracellular cell-pathogen communication is mediated by proteins.

## Pathogen multiplicity, diversity, and replication dynamics

The study of infected host cells using single-cell technologies provides high-resolution insight into several aspects of intracellular pathogen replication. First, information about pathogen burden and production for each cell can be assessed, revealing differential contributions of host cell subsets to pathogen propagation or presentation of antigens to the immune system. Second, diversity among pathogen populations replicating within a host are readily quantified, including within distinct cell populations. Pathogen diversity is important because varied genomic sequences or functional properties may enhance replication fitness and immune evasion capability. And third, the dynamics of pathogen assembly and replication by necessity require monitoring discrete steps in the pathogen life cycle within individual cells. Recent advances in our understanding of these three themes, pathogen multiplicity, diversity, and assembly dynamics have become possible because of single-cell technologies.

The number of virions or bacteria that establish infection within a cell can influence downstream events in the pathogen’s life cycle or the infected host cell, with potential consequences for the organism at large. Viral recombination, persistent latent infection, pathogen-mediated cytopathicity, and antigen presentation to the immune system, for example, may be impacted by cellular pathogen burden. In a recent study of influenza mRNA copies within single dendritic cells, qRT-PCR revealed greater multiplicity of infection among a specific subset of dendritic cells: those residing in the respiratory tract, compared to their lymph node-resident counterparts^44^. Identification of antigen presenting cells with higher pathogen burden may implicate such cells in regulating adaptive immune responses. Single-cell approaches have also yielded renewed estimates of the size of viral reservoirs and cellular burst size, due to the improved accuracy of more sensitive and efficient assays^45–48^, as discussed below.

### Laser-capture and microdissection

One such approach measured DNA copy number per cell by laser-capture microdissection (LCM) followed by qPCR to investigate the distribution of sensory neurons for latent HSV-1 and VZV infections^48^. LCM is a technology that cleanly “picks” a small cluster of material, as little as one cell, from an appropriately preserved tissue specimen^49^. Subsequent molecular analyses can identify the cell(s) picked, their infection state, and their phenotypes. Infected DNA PCR-positive cells were found approximately ten times more frequently by this method than by the conventional ISH method, indicating a much more pervasive latent reservoir in neuronal cells than previously appreciated. In addition, the HSV-1 and VZV DNA burden was determined to be ~10 and 7 copies per positive cell, respectively.

Similarly, applying RNA- and DNAscope to the study of SIV-infected cells in rhesus macaques revealed broad distribution of RNA- and DNA-positive cells throughout the body at all stages of SIV infection, again highlighting the tremendous infected cell burden^45^. It was also noted that many T-cells residing in lymph nodes harbored two distinct SIV proviral DNA punctae within their nuclei during acute infection, consistent with superinfection of those cells. This is a key mechanism underlying recombination of HIV, leading to increased pathogen diversification and immune escape. Another novel application of single-cell virus quantitation was the study of cellular multiplicity of infection dynamics during virus colonization of a plant host, revealing spatio-temporal relationships of viral spread among cells with varying burden^50^. These and other assays to quantify single-cell viral copy numbers and the frequency of cells positive for viral genomes have been developed successfully for multiple viruses^34,51^.

To understand the relative contribution of an infected cell to pathogen replication within the host, quantitation of the burst size or microbial output per cell is required. Some cell types or cell subsets may exhibit greater propensity to generate high quantities of viral progeny than others, for example (Fig. 1). Using in-situ hybridization combined with tyramide signal amplification and enzyme-linked fluorescence to enumerate SIV virions in macaque specimens during acute infection, four times as many virions were observed surrounding activated CD4 T-cells compared to a resting cell: mean counts of 191 and 34 virions per cell, respectively^47^. However, due to a longer half-life and greater prevalence, resting cells are estimated to produce 70% of virus in acute infection. Other studies have examined the impact of suppressive antiretroviral therapy on viral RNA expression by HIV-1-infected cells in patient lymph nodes. Viral genomic RNA qRT-PCR analysis of individual infected cells isolated at limiting dilution revealed a relatively constant mean RNA content per cell of around 5000 copies^46^, regardless of viral load and suppressive treatment status. Taken together, these snapshots of virion production reveal potential cell-derived sources of differential HIV/SIV replication as well as sources of viral persistence in the face of treatment.

Pathogens employ multiple strategies to evade immune recognition and develop resistance to therapeutic drugs. Genetic diversity is one such strategy and results in pathogen subpopulations or quasispecies equipped to overcome drug or immune pressure targeting susceptible genes. Early efforts to study HIV-1 sequence diversity with single-cell resolution employed fluorescence ISH to estimate the number of DNA proviruses in individual splenocytes. This work revealed an average of 3–4 per cell. These cells were then subjected to LCM of HIV-positive nuclei for PCR and sequencing^52^. Multiple proviruses within a cell contained genetically distinct sequences, fulfilling a key requirement for viral recombination to occur in progeny virus. More recent analysis of HIV-1 DNA copy number and genetic sequence variation in blood and lymph node showed that most infected cells contain only one copy of HIV-1 DNA^53,54^, and therefore viral recombination potential may be more limited in virus produced by these cells. In a related effort to determine hepatitis C virus (HCV) diversity among infected cells in vitro, single-cell RNA viral loads were measured by qRT-PCR followed by sequencing of the subgenomic amplicon^55^. On average, cells contained ~100 HCV RNA copies each and viral quasispecies varied extensively between cells, consistent with cellular compartmentalization of viral RNA sequences and independent evolution within cells. Finally, single cell-associated VSV RNA sequence analysis highlighted two additional mechanisms for generating viral diversity: co-transmission of different viral genomes within an infectious unit to an individual cell and variable spontaneous virus mutation rates across cells^56^. Combined, these analyses identify multiple sources of viral diversity within a cell, including heterogeneity among virus populations entering the cell, such as HIV and SIV, as well as viral evolution during intracellular replication, observed for HCV and VSV. Such mechanisms increase the potential for viral escape and recombination, likely favoring pathogen resistance to host immunity.

Intracellular bacterial pathogens also display host cell-to-cell heterogeneity, which can have important implications for how the host cell responds to infection. In two recent examples using macrophages infected by *Salmonella tryphimurium*, subpopulations of bacteria were identified that differ in their effect on the host cell. First, using a fluorescence-based approach, many macrophage-internalized bacteria were observed to not replicate but rather entered a dormant-like state in host cells^57^. Development of this nonreplicating subpopulation, which is able to persist in the face of antibiotics, was subsequently found to be regulated by the host cell vacuolar environment^58^. Second, using scRNA-seq to profile host and bacterial transcripts, type I IFN responses in macrophages were found to be modulated by variable levels of transcription factor expression in bacterial subpopulations^59^. These studies highlight the dynamic nature of pathogen-host cell encounters and the potential for increased heterogeneity and subpopulation formation during these interactions.

Microorganism replication is often a highly orchestrated, multi-step process that evolved to favor pathogen replicative fitness and survival. Studying these processes in single cells offers the greatest resolution to delineate the sequence of events and elucidate mechanisms that govern the life cycle kinetics. A recent example sought to understand the benefit to the virus of HIV-1’s active inhibition of the pace of virion assembly^60^. Combining microscopy, FACS, and labeled virus technology the viral molecular mechanism mediating the delay of virion assembly by HIV-1 was identified; the duration of the delay is consistent with the time required for the degradation of host restriction factors. Thus, by slowing HIV-1 virion formation, deleterious host proteins could be excluded from budding particles. In another example, influenza A virus (IAV) entry and gene segment localization was assessed using an RNA labeling approach termed padlock probes^61^. The high specificity and multiplexing capability of this approach enabled detection of all eight IAV vRNA gene segments within a cell, definition of infectious stages across cell populations, and determination that productive IAV cell co-infections required for genome reassortment are temporally limited by the replication stage of the primary infection. These findings provide potential mechanistic insight into the low reassortment frequencies observed for human IAV infections. Such studies are possible through the combination of single-cell imaging, molecular, and flow cytometric approaches. Further, scRNA-seq of more complex pathogens such as *Plasmodium*, which exhibit multiple life cycle stages in different hosts, unsurprisingly revealed discrete signatures associated with pathogenic and sexual vs. asexual stages^62,63^. In addition to the known master regulator of sexual development, AP2-G, several other regulators of gene expression were identified in sexually committed parasites. Previous challenges in distinguishing the subpopulation of sexually committed AP2-G+ parasites from their otherwise visually similar asexual counterparts were overcome by scRNA-seq detection of this key transcription factor.

## Conclusions

It is no longer tenable to study the complexity of the immune system without using single-cell technologies; the heterogeneity of the immune system and its interconnected elements is simply too great. To address this need, a wide variety of single-cell technologies are now available for the study of host–pathogen interactions. High-resolution single-cell imaging, both in vitro and in vivo, provides information about the organization of single cells as they interact during infection or as the host immune response is invoked. In vivo imaging has the added advantage of providing dynamic information, as investigators can witness single cells moving through tissue or changing over time. Cytometry—using fluorescent tags (FACS) or elemental tags (CyTOF)—is now capable of measuring expression of 30+ proteins simultaneously, providing detailed phenotypic and functional analysis of host cells before and after infection, or as they participate in immune responses. Fluorescence cytometry is crucial for the isolation of individual viable cells so that the immune responses and intracellular communication can be deconstructed and analyzed individually. Recently developed application technologies provide for measurement of mRNA within the cell (albeit with a limited number of targets). Molecular assays often provide the most information about hosts and pathogens, and are particularly useful for identifying infected cells or characterizing pathogens (since intracellular pathogens are often too small for detection by protein expression). With the recent emergence of single-cell RNA-sequencing, the information content available using these approaches is remarkable.

However, one should never lose sight of the immense amount of post-transcriptional regulation and selective trafficking of proteins that impact cellular responses: molecular quantification is still limited. Thus, in many cases, the most powerful approaches will combine multiple technology modalities to provide a comprehensive analysis of infected cells and immune responses. As these new approaches are created and used, new drug and vaccine strategies are likely to emerge, alongside a better understanding of disease pathogenesis.
